# Wixela Inhub: Dosing Performance *In Vitro* and Inhaled Flow Rates in Healthy Subjects and Patients Compared with Advair Diskus

**DOI:** 10.1089/jamp.2019.1584

**Published:** 2020-12-02

**Authors:** Andrew Cooper, James Parker, Mark Berry, Róisín Wallace, Jon Ward, Richard Allan

**Affiliations:** ^1^ Mylan Pharma UK Ltd., Sandwich, United Kingdom.; ^2^ Mylan, Dublin, Ireland.

**Keywords:** asthma, COPD, dry powder inhaler, flow rate

## Abstract

***Background:*** Wixela™ Inhub™ is a fluticasone propionate/salmeterol dry powder inhaler developed as a generic equivalent of Advair Diskus^®^ for the treatment of asthma and chronic obstructive pulmonary disease (COPD). Wixela Inhub and Advair Diskus are comparable in terms of functionality, user interface, and device resistance. The primary objectives of the studies were to evaluate *in vitro* dose delivery with Wixela Inhub compared with Advair Diskus at relevant flow rates and to explore inhalation profiles generated by patients with asthma or COPD.

***Methods:*** *In vitro studies:* Emitted dose (ED) and individual dose aerodynamic particle size distribution (APSD) were measured at flow rates ranging from 30 to 90 L min^−1^. *Patient inhalation study:* Inhalation profile recording was conducted three times in each patient (40 children with asthma, 14 adults with asthma, and 14 adults with severe-to-very-severe COPD) with an empty Inhub in an open-label study. The primary endpoint was peak inhaled flow rate (PIFR). An additional endpoint was peak pressure drop.

***Results:*** *In vitro studies:* ED and APSD delivered from Wixela Inhub showed low flow dependency across the patient-relevant flow-rate range. Wixela Inhub gave *in vitro* performance comparable with Advair Diskus for all strengths and flow rates. *Patient inhalation study:* For Inhub, mean PIFR was lowest for children with asthma ages 4 to 7 years (50.6 L min^−1^) and highest for adults with asthma (74.8 L min^−1^). For adults with severe-to-very-severe COPD, mean PIFR was 69.5 L min^−1^ with Inhub. The PIFRs observed with Diskus were higher than those with Inhub, consistent with slightly higher resistance measured *in vitro*. The difference in resistance did not impact demonstration of bioequivalence and does not impact substitutability of the product. Peak pressure drop values were comparable between Diskus and Inhub.

***Conclusions:*** Comparable *in vitro* performance of Wixela Inhub to Advair Diskus confirmed that Wixela Inhub is a generic equivalent to Advair Diskus across all patient groups.

## Introduction

Wixela™ Inhub™ is a fluticasone propionate (FP)/salmeterol (as xinafoate salt) dry powder inhaler (DPI) developed in three strengths (100/50 mcg, 250/50 mcg, and 500/50 mcg) approved by the U.S. Food and Drug Administration (FDA) as a generic equivalent of Advair Diskus^®^ for the treatment of asthma and chronic obstructive pulmonary disease (COPD).^(1)^ Advair Diskus is a DPI that delivers fixed-dose combinations of FP and salmeterol.^(2)^ The Inhub and Diskus devices each hold multiple (60) doses of medication, premetered, and stored as individual doses.

The high prevalence and cost of asthma and COPD contribute to a substantial patient burden.^(3)^ Generic products have been developed in an attempt to lessen this financial burden, thereby improving patient access and adherence. Establishing bioequivalence (BE) for locally acting drug products, such as DPIs, has proved challenging from a technical and regulatory perspective. As a result, before the approval of Wixela Inhub, no generic DPIs were available to patients in the United States. In U.S. regulation, BE is defined as “the absence of a significant difference in the rate and extent to which the active ingredient or active moiety in pharmaceutical equivalents or pharmaceutical alternatives becomes available at the site of drug action when administered at the same molar dose under similar conditions.”^(4)^

The FDA's approach to BE for orally inhaled products is derived from the understanding that BE at the site of local drug action cannot be demonstrated by systemic pharmacokinetic (PK) studies alone, leading to the development of a “weight-of-evidence” approach to the demonstration of BE, involving an extensive package of clinical, PK, and *in vitro* data.^(5)^ This approach was detailed in draft product-specific guidance for FP and salmeterol xinafoate inhalation powder issued in 2013,^(6)^ and was the pathway to approval followed for Wixela Inhub. The *in vivo* BE studies carried out for Wixela Inhub are described elsewhere.^(7,8)^

The *in vitro* studies recommended to establish BE comprise single actuation content (SAC) and aerodynamic particle size distribution (APSD), each determined at flow rates of 30, 60, and 90 L min^−1^ and at multiple life stages of the product. Statistical comparison of test and reference is carried out using population bioequivalence (PBE) analysis of the SAC and the impactor-sized mass (ISM) derived from the APSD data (ISM is defined as the sum of drug mass on all impactor stages except the top stage). The use of PBE analysis is a consistent feature of the FDA's recommendations for assessment of *in vitro* BE, a key feature of which is that the difference between test and reference variance is included in the calculation.^(9)^ Cascade impactor profiles, together with mass median aerodynamic diameter (MMAD), geometric standard deviation (GSD), and fine particle mass (FPM), are also submitted as supportive evidence of APSD equivalence.

In addition to *in vitro* and *in vivo* studies, the FDA guidance also includes formulation and device requirements for BE. Wixela Inhub contains a qualitatively and quantitatively equivalent powder formulation to Advair Diskus. The Inhub device complies with FDA recommendations for similarity of critical device features, including format and number of doses, size and shape, external operating principles, resistance, and the incorporation of a dose counter.

**FIG. 1.**
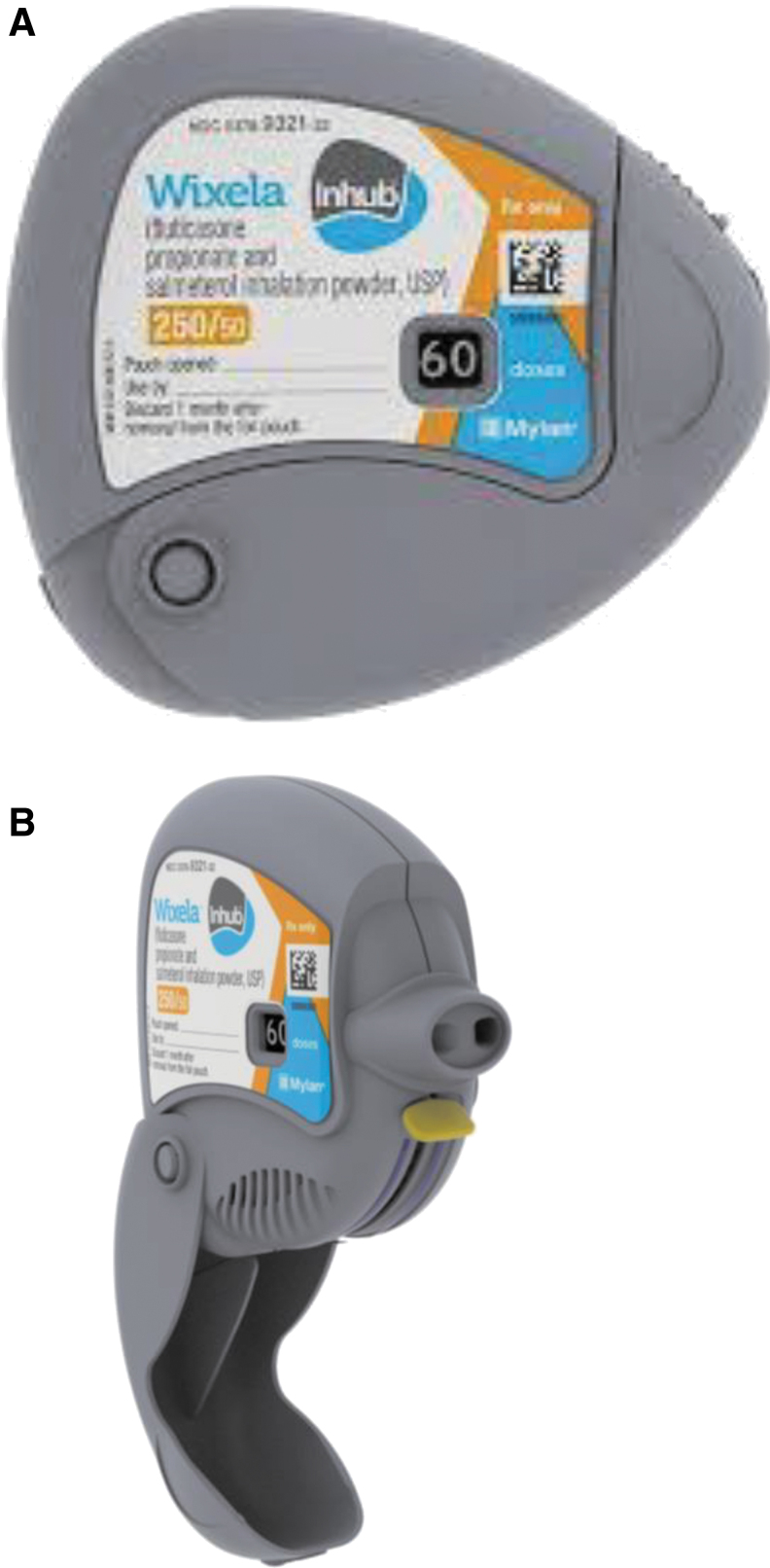
**(A)** External view of Wixela™ Inhub™ Inhaler **(B)** Open View of Wixela Inhub Inhaler. Inhub includes a mouthpiece cover, a lever, and a dose counter.

Inhub is similar in design to Diskus. Both devices include a mouthpiece cover, a lever, and a dose counter. Inhub, as shown in Figure 1A (closed view) and B (open view), is a small, handheld inhalation device of similar size and shape to Diskus. The usage of Inhub is very similar to that of Diskus. Inhub is operated by completing five key steps: (i) open the mouthpiece, (ii) push down a lever, (iii) inhale, (iv) close the mouthpiece, and (v) rinse your mouth.

DPIs such as Diskus and Inhub rely on the patient's inspiratory effort to deliver and deaggregate the powder to generate the lung-deposited dose. As a result, it is important to characterize the flow rates that patients achieve through the inhaler in relation to the flow rates required to deliver an efficacious dose. Patient flow rates are determined by the interaction between their inspiratory capabilities (which may be impaired by their age and/or disease state) and the airflow resistance of the inhaler. The resistance of the Diskus inhaler has typically been classified as “medium.”^(10,11)^ In relation to the use of the In-Check DIAL G16 (Clement-Clarke International Ltd, Harlow, England) inspiratory flow assessment device, Sanders used a 5-point resistance classification in which Diskus is classified “medium-low” resistance.^(12)^

A primary objective of the present studies was to assess the inhalation flow profiles of healthy subjects and, in particular, patients with asthma or COPD, through the Inhub inhaler to demonstrate that the population of patients likely to use Inhub (children and adults with asthma and adults with moderate-to-severe COPD) can generate sufficient flow to aerosolize drugs contained in it. Additional primary objectives were to evaluate the *in vitro* device resistance of the Inhub and Diskus devices, and to evaluate the *in vitro* dose-delivery characteristics of Wixela Inhub at clinically relevant flow rates and compare these characteristics to those achieved for Advair Diskus to demonstrate *in vitro* BE.

## Materials and Methods

### *In vitro* studies of dose delivery

#### Emitted dose

Emitted dose (ED) was measured at various flow rates using USP<601> Apparatus B (Dosage Unit Sampling Apparatus, Copley, Nottingham, United Kingdom). A vacuum pump and a critical flow controller (Copley TPK) were used. The flow rate was measured as described in USP<601>, using a flow meter calibrated for the volumetric flow leaving the meter (Copley DFM or equivalent). Flow duration was set to give a volume of 2 L. The ED was recovered from the apparatus with a methanol buffer—diluent and was analyzed by liquid chromatography.

#### Aerodynamic particle size distribution

APSD of individual doses was measured at various flow rates, using USP<601> Apparatus 5 (Next Generation Impactor™ [NGI], Copley, Nottingham, United Kingdom). A vacuum pump and a critical flow controller (Copley TPK) were used. The flow rate was measured as described in USP<601>, using a flow meter calibrated for the volumetric flow leaving the meter (Copley DFM3 or equivalent). Flow duration was set to give a volume of 4 L. The FP and salmeterol were recovered from the NGI stages and accessories (mouthpiece adapter/induction port and preseparator) with a methanol—buffer diluent and were analyzed by liquid chromatography.^(13)^ Deposition on individual impactor stages and accessories was determined. Mass balance (sum of mass determined on all stages and accessories expressed as a percentage of the product target ED), ISM (sum of mass determined on stages 2 through 7 and micro-orifice collector), FPM (sum of mass <5 μm estimated by interpolation), MMAD, and GSD were reported.

#### Measurement of airflow resistance

Airflow resistance was measured using USP<601> Apparatus B. A dose was fired to waste, after which the pressure drop across the device at flow rates in the range of 20 to 100 L min^−1^ was measured by connecting a manometer to the pressure tap P1 on the apparatus. The specific airflow resistance, R in kPa^0.5^ L^−1^ minutes, was estimated by linear regression as the slope of the relationship between pressure √P_d_ (where P_d_ is measured in kPa) and volumetric flow rate (Q, in L min^−1^); that is, according to the equation √P_d_ = RQ.^(14)^

For Inhub, the airflow resistance was measured for one dose from each of five devices of each product strength. For Diskus, the airflow resistance was measured for five doses from each of nine devices of the high and low strengths.

In addition, the variability of the resistance of each device was assessed by making a larger number of measurements of pressure drop at 60 L min^−1^ and of flow rate at 4 kPa across multiple devices and product batches. For Inhub, 5 to 6 devices from each of 13 product batches were tested at 60 L min^−1^. For Diskus, 3 devices from each of 6 product batches were tested at 60 L min^−1^. For both devices, the flow rate at 4 kPa pressure drop was measured for 20 devices from each of 9 product batches.

### Patient inhalation flow profile study

#### Study design and conduct

The primary objective of the multicenter, open-label, 2 × 2 crossover study conducted in healthy volunteers and patients with asthma (including children) or COPD was to measure the inhalation flow rates generated when using Inhub and Diskus DPIs. The study protocol and other relevant study documentation were reviewed and approved by applicable independent Ethics Committees or Institutional Review Boards, and the study was conducted in accordance with the requirements of the Declaration of Helsinki and applicable local regulatory requirements.

#### Study participants

**FIG. 2.**
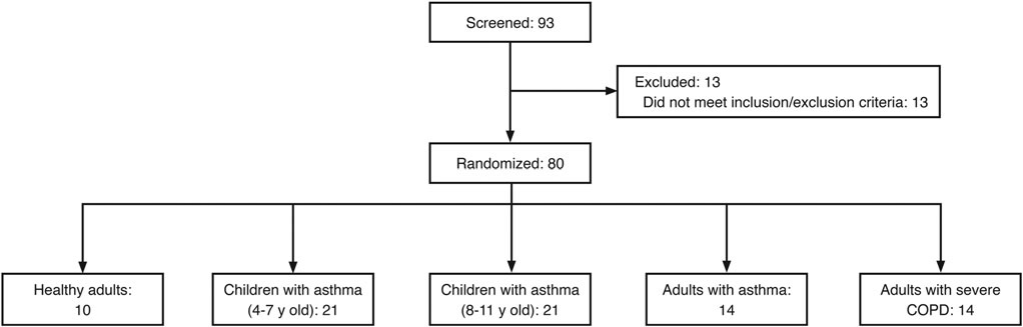
Subject disposition. A total of 93 patients were screened and 80 were enrolled from August through December 2013 at four study sites in Germany. Seventy-eight subjects were included in the inhalation analysis data set. COPD, chronic obstructive pulmonary disease.

The total sample size for this study (*n* = 78) was based on the number of subjects in each group needed to achieve ≥80% probability that the 10th, 50th, and 90th percentiles for the primary endpoint were estimated reliably (±10 L min^−1^ of the true values). Enrolled subjects were segmented into five groups, each including both male and female participants, with the following inclusion criteria (Fig. 2):

- Healthy adults (*n* = 10)
- ○ Ages 18 to 60 years
- ○ Forced expiratory volume in 1 second (FEV_1_) and forced vital capacity (FVC) ≥80% of the predicted value for their age, height, sex, and race
- ○ Nonsmokers or ex-smokers who had a history of ≤10 pack years and had stopped smoking ≥6 months before the screening visit
- ○ Subjects were excluded if they had a history of chronic airway disease or current evidence of upper or lower respiratory tract infection
- Children with asthma were enrolled into one of two groups, according to age (ages 4–7 years [*n* = 20] or ages 8–11 years [*n* = 20])
- ○ Established diagnosis of asthma (controlled or partly controlled) for ≥6 months
- ○ FEV_1_ ≥ 60% of the predicted value for their age, height, sex, and race
- ○ Either used asthma medication as regular maintenance therapy for ≥3 months before study screening; or demonstrated reversibility, defined as a postbronchodilator increase in FEV_1_ of ≥10% at screening or within previous 12 months
- Adult subjects with asthma (*n* = 14)
- ○ Ages 18 to 80 years
- ○ Established diagnosis of asthma (controlled or partly controlled) for ≥6 months
- ○ Had a prebronchodilator FEV_1_ of ≤60% predicted and showed reversibility at the screening visit (i.e., postbronchodilator FEV_1_ increased ≥12% and 200 mL)
- ○ Were required to have received regular treatment with an inhaled asthma medication at a consistent dosage for ≥4 weeks before screening
- ○ Smoking history ≤10 pack years
- Adult subjects with COPD (*n* = 14)
- ○ Ages 40 to 80 years
- ○ Diagnosed with severe COPD, defined as a postbronchodilator FEV_1_/FVC ratio of <0.7 and postbronchodilator FEV_1_ ≥ 30% and <50% of predicted
- ○ Received regular treatment with any COPD medication at a consistent dosage for ≥4 weeks before screening
- ○ Required to be either current or ex-smokers, with a smoking history ≥10 pack years.

##### Exclusion criteria

Exclusion criteria for study participation included the following:

- For all subjects with asthma or COPD, participants were excluded if they had experienced an acute exacerbation of their asthma or COPD or if they had been hospitalized or visited the emergency department due to an exacerbation within 2 months before study enrollment
- A neurologic disease that affected neuromuscular function/performance or respiratory muscles (e.g., myasthenia gravis, amyotrophic lateral sclerosis), or a history of life-threatening asthma episodes or significant lung/lower respiratory tract diseases (other than asthma or COPD)
- Acute or chronic airway infections within 30 days of screening
- Pregnant or lactating women (ages ≥18 years) and postmenarchal girls (ages ≤11 years).

These study groups were selected because they covered the anticipated range of subjects (in particular, age and maximum disease severity) in whom the device will be used. A number of clinical parameters differ in these populations, including age, body weight and height, and severity of disease, which can influence inspiration.^(15–17)^ Findings from these study groups also enabled comparison to healthy subjects to justify their use in the PK BE studies. All study subjects provided written informed consent. The parents or legal guardians of the enrolled children provided written informed consent, and each child provided written informed assent.

#### Assessments

##### Inhalation Profile Analyser

The Inhalation Profile Analyser (IPA) was a bespoke, validated, computer-driven system designed to record the inhalation profile (inspiratory flow rate and pressure drop as a function of time) that a subject generates when inhaling through an inhaler.

The system was formed of three main components:

1. An inhaler connecting box (ICB) that contained either the Inhub or the Diskus inhaler
2. An inhalation profile recorder (IPR)
3. A laptop with software designed to facilitate measurement of inhalation profiles.

The ICB was a plastic box used to connect the inhaler under investigation to the IPR through a length of tubing. An empty inhaler was placed inside the ICB and the ICB was sealed, so that the inhaler was *not* accessible to either the investigator staff or the subject. This means the inhaler could not be opened or closed and the dose-loading mechanism could not be engaged; the only way the subject could interact with the (empty) inhaler was to inhale air through it. The use of the ICB also meant subjects were unaware of the inhaler through which they were inhaling at the time. The investigator was not blinded, as they would see which inhaler was being studied on the software used to capture and analyze the data. Subjects inhaled through the inhaler using a disposable filter (Vitalograph BV Filter 2820, Maids Moreton, United Kingdom, with a reported resistance of 0.097 kPa/L/s) to prevent cross contamination.

The IPR contained a pressure transducer that measures the pressure drop (in kPa) created at the mouthpiece of the device when a subject inhaled through it. Measurements were taken every 100 milliseconds, allowing a plot of pressure drop against time to be constructed; this plot is termed the inhalation profile. The pressure transducer was calibrated against an external source to ensure the pressure drops measured were valid.

The software enabled the conversion of the pressure data to provide outputs such as peak inhaled flow rate (PIFR) and peak pressure drop; these data were then recorded accordingly.

##### Study visits and procedures

Subjects attended a screening visit and an inhalation profile testing visit that was conducted 0 to 21 days after screening for healthy adults and children, or 1 to 14 days after screening for adults with asthma or COPD. The inhalation visit was followed 1 to 7 days later with a telephone follow-up.

During the inhalation profile testing visit, the IPA was used to measure, record, and graphically represent the inhalation profiles generated by inhaling through the DPIs. Inhub (test) and Diskus (reference) devices were connected to the IPA using an ICB. As the ICB enclosed the devices, subjects did not know which device they were using. Because this study was designed to assess only inhalation profiles, neither the Inhub nor the Diskus devices contained medication or powder excipients. The Inhub devices contained a blank disk, as this forms part of the airflow path of the device, and the Diskus devices had the blister strips removed, as these do not form part of the airflow path of the device; therefore, the performance of the devices would be unaffected by these modifications.

To generate each inhalation profile, subjects inhaled through the DPI within the ICB through a disposable filter. Before the inhalation profiles were obtained, the IPA was used to train subjects to inhale rapidly and deeply using both the Inhub and Diskus devices. After the training inhalations, each subject was asked to generate three inhalation profiles through one device, followed by three inhalation profiles through the other device. The order in which the devices were used was determined by a preset randomization code.

#### Study endpoints

The primary endpoint was PIFR (L min^−1^) generated through each DPI. The data were assessed in two ways: by calculating the mean of the three values obtained, and by reporting the highest value obtained.

Peak pressure drop generated through each DPI (kPa) was a further endpoint. The data were assessed in two ways: by calculating the mean of the three values obtained, and by reporting the highest value obtained.

#### Statistical analyses

##### *In vitro* studies of dose delivery

PBE analysis of ED (SAC) and ISM data generated at 30, 60, and 90 L min^−1^ were carried out as recommended by the FDA.^(6,18)^ Statistical analyses were conducted using SAS software version 9.3 (Cary, NC). Each flow rate and device life stage (beginning, middle, and end for SAC; beginning and end for ISM) was subject to separate PBE analysis for each endpoint and active ingredient, giving 90 comparisons in total across the three product strengths.

##### Airflow resistance measurements

Statistical analysis of airflow resistance measurements was carried out using Microsoft Excel 2016 (Redmond, WA). Mean values for specific airflow resistance of each device were calculated. Mean, relative standard deviation, and range values were calculated for pressure drop at 60 L min^−1^ flow rate and flow rate at 4 kPa pressure drop.

##### Patient inhalation flow profile study

All endpoints were analyzed for each subject group and device separately, using summary statistics. Statistical analyses were conducted using SAS software version 9.3 and the IPA system, which generated summary statistics for the endpoint.

## Results

### *In vitro* studies

#### ED and APSD at 30 to 90 L min^−1^ flow rates

**FIG. 3.**
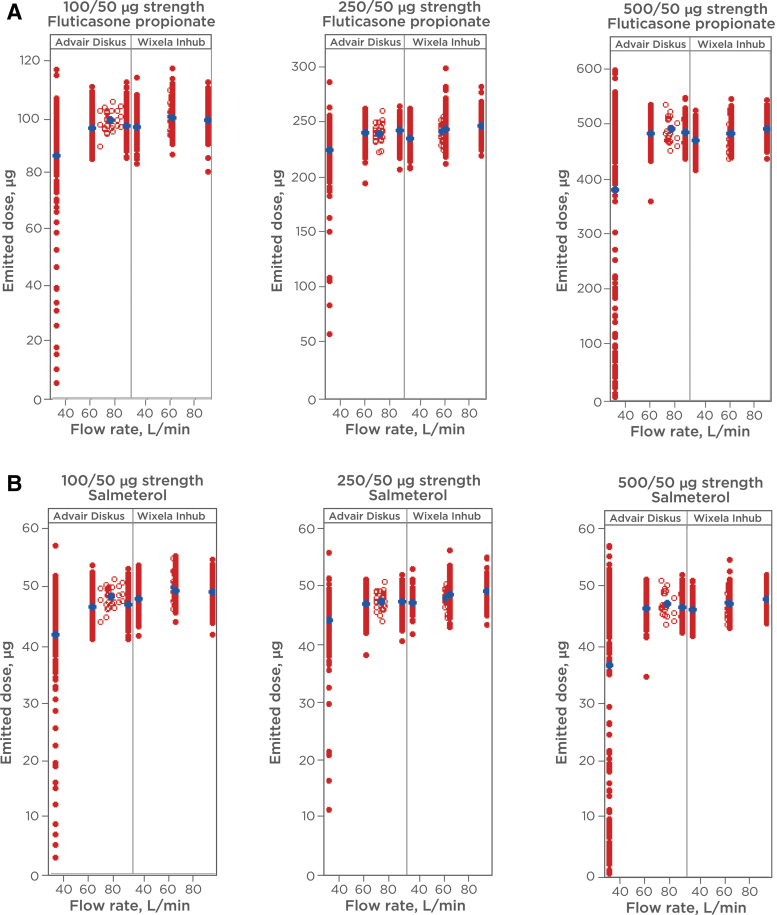
**(A)** ED of FP from Wixela™ Inhub™ and Advair Diskus^®^. ED was measured at various flow rates. Flow duration was set to give a volume of 2 L. The ED was recovered from the apparatus with a methanol—buffer diluent and analyzed by liquid chromatography. *Red circles* show individual measurements (closed = 30, 60, 90 L min^−1^; open = 4 kPa). *Blue circles* show mean values. **(B)** ED of Salmeterol from Wixela Inhub and Advair Diskus. ED was measured at various flow rates. Flow duration was set to give a volume of 2 L. The ED was recovered from the apparatus with a methanol—buffer diluent and analyzed by liquid chromatography. *Red circles* show individual measurements (closed = 30, 60, 90 L min^−1^; open = 4 kPa). *Blue circles* show mean values. ED, emitted dose; FP, fluticasone propionate.

**FIG. 4.**
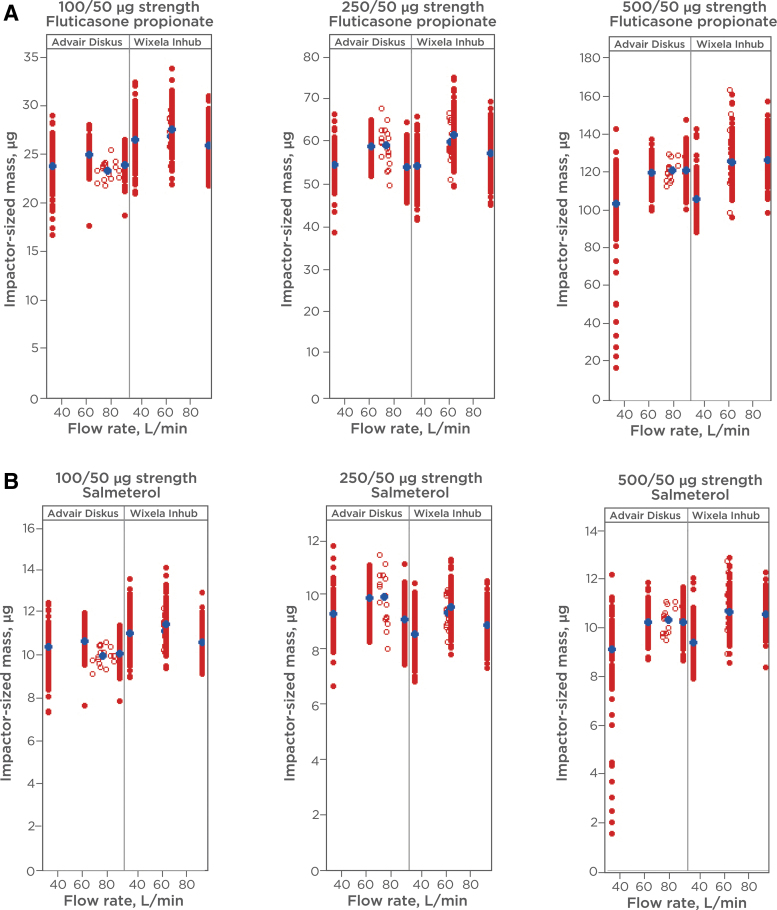
**(A)** ISM of FP from Wixela Inhub and Advair Diskus. ISM of individual doses was measured at various flow rates. Flow duration was set to give a volume of 4 L. The aerodynamic fractions were recovered from the NGI stages with a methanol—buffer diluent and were analyzed by liquid chromatography. *Red circles* show individual measurements (closed = 30, 60, 90 L min^−1^; open = 4 kPa). *Blue circles* show mean values. **(B)** ISM of salmeterol from Wixela Inhub and Advair Diskus. ISM of individual doses was measured at various flow rates. Flow duration was set to give a volume of 4 L. The aerodynamic fractions were recovered from the NGI stages with a methanol—buffer diluent and were analyzed by liquid chromatography. *Red circles* show individual measurements (closed = 30, 60, 90 L min^−1^; open = 4 kPa). *Blue circles* show mean values. ISM, impactor-sized mass; NGI, Next Generation Impactor™.

ED (described as “SAC” in FDA product-specific guidance)^(6)^ and APSD were determined at flow rates of 30, 60, and 90 L min^−1^ for determination of *in vitro* equivalence between Wixela Inhub and Advair Diskus. The products were tested across device life stages (beginning, middle, and end life stages for ED; beginning and end life stages for APSD). The results from the *in vitro* studies are illustrated in Figures 3A, B and 4A, B.

Comparable *in vitro* performance was observed across the flow rates tested for both parameters in all product strengths, with a low degree of flow dependency observed in both products. The largest differences in mean values between products were observed at the lowest flow rate of 30 L min^−1^. Despite similarity in the majority of the data, poor dose emission was observed from a significant minority of the Diskus devices at this flow rate, resulting in lower mean values for SAC in all three strengths.

PBE analyses of the SAC and ISM data are presented in Tables 1–3 and Tables 4–6, respectively. For individual comparisons, PBE was established if the 95% upper confidence bound of the linearized criteria was ≤0.^(18)^ This acceptance criterion was met for 89 of the 90 PBE comparisons for the two parameters across the strength, and is a high success rate considering the multiplicity of the comparisons. The single comparison (FP ISM 30 L min^−1^, beginning of life in the 100/50 mcg strength), for which the upper confidence bound fell marginally above the PBE acceptance limit, was for a flow rate and life stage where Advair Diskus shows higher variability and lower mass balance.

**Table 1.** Population Bioequivalence Statistics for Single Actuation Content (mcg): 100/50 mcg Strength

Flow rate (L/min)	Active	Life stage	GM (T)	GM (R)	SD (T)	SD (R)	Linearized criteria	
							Point estimate	95% Upper confidence bound
30	FP	BoL	95.23	66.14	4.34	22.64	–0.8965^R^	–0.6246
		EoL	95.11	88.74	4.34	12.88	–0.0681^R^	–0.0489
		MoL	95.93	92.93	5.48	8.02	–0.0245	–0.0215
	Sal	BoL	47.52	32.16	1.79	11.08	–0.8877^R^	–0.6107
		EoL	47.73	43.17	2.15	6.22	–0.0626^R^	–0.0427
		MoL	47.93	45.19	2.59	4.01	–0.0228	–0.0189
60	FP	BoL	97.46	89.67	3.43	3.04	–0.0139	–0.0120
		EoL	99.30	99.74	4.08	5.28	–0.0221	–0.0211
		MoL	99.91	96.34	6.02	3.29	–0.0172	–0.0153
	Sal	BoL	48.70	43.73	1.74	1.55	–0.0093	–0.0068
		EoL	49.39	48.48	1.93	2.54	–0.0219	–0.0207
		MoL	49.51	46.78	2.70	1.53	–0.0158	–0.0137
90	FP	BoL	96.72	90.76	4.05	3.26	–0.0163	–0.0144
		EoL	97.73	101.42	4.14	3.97	–0.0192	–0.0179
		MoL	99.29	95.99	5.30	3.41	–0.0182	–0.0166
	Sal	BoL	48.50	44.36	1.94	1.50	–0.0124	–0.0102
		EoL	48.83	49.37	2.04	1.61	–0.0201	–0.0193
		MoL	49.43	46.76	2.37	1.54	–0.0166	–0.0148

Reference-scaled PBE criteria used if denoted by ^R^, otherwise constant-scaled criteria used.

BoL, beginning of life; EoL, end of life; FP, fluticasone propionate; GM, geometric mean; MoL, middle of life; PBE, population bioequivalence; R, reference; Sal, salmeterol; SD, standard deviation; T, test.

**Table 2.** Population Bioequivalence Statistics for Single Actuation Content (mcg): 250/50 mcg Strength

Flow rate (L/min)	Active	Life stage	GM (T)	GM (R)	SD (T)	SD (R)	Linearized criteria	
							Point estimate	95% Upper confidence bound
30	FP	BoL	231.06	195.91	8.51	39.73	–0.2410	–0.1708
		EoL	235.25	229.26	8.14	20.73	–0.0796	–0.0467
		MoL	236.66	239.46	9.75	11.87	–0.0271	–0.0199
	Sal	BoL	46.76	38.61	1.44	7.86	–0.0284	–0.0256
		EoL	47.47	45.11	1.23	4.07	–0.0216	–0.0205
		MoL	47.47	47.10	1.53	2.36	–0.2343^R^	–0.1623
60	FP	BoL	240.25	229.60	11.98	7.12	–0.0269	–0.0235
		EoL	241.73	246.77	9.61	9.90	–0.0224	–0.0215
		MoL	244.18	240.70	13.11	8.73	–0.0175	–0.0159
	Sal	BoL	48.21	45.37	1.66	1.48	–0.0207	–0.0197
		EoL	48.61	48.51	1.53	1.93	–0.0192	–0.0179
		MoL	48.90	47.30	2.23	1.78	–0.0171	–0.0156
90	FP	BoL	242.21	232.07	8.85	8.15	–0.0217	–0.0211
		EoL	245.03	249.79	8.36	5.49	–0.0192	–0.0179
		MoL	249.78	242.85	10.60	7.89	–0.0190	–0.0178
	Sal	BoL	48.65	45.73	1.60	1.69	–0.0198	–0.0192
		EoL	49.06	49.07	1.52	1.10	–0.0194	–0.0183
		MoL	49.61	47.64	1.98	1.56	–0.0174	–0.0159

Reference-scaled PBE criteria used if denoted by ^R^, otherwise constant-scaled criteria used.

R, reference; Sal, salmeterol; T, test.

**Table 3.** Population Bioequivalence Statistics for Single Actuation Content (mcg): 500/50 mcg Strength

Flow rate (L/min)	Active	Life stage	GM (T)	GM (R)	SD (T)	SD (R)	Linearized criteria	
							Point estimate	95% Upper confidence bound
30	FP	BoL	458.99	104.24	18.06	162.57	–2.0700^R^	–0.7543
		EoL	470.36	481.88	19.97	55.44	–0.0567^R^	–0.0421
		MoL	452.55	445.30	20.27	76.15	–0.1388^R^	–0.1043
	Sal	BoL	46.20	10.40	1.47	16.02	–2.0144^R^	–0.7032
		EoL	47.38	47.41	1.72	5.38	–0.0564^R^	–0.0423
		MoL	45.55	43.75	1.68	7.42	–0.1337^R^	–0.1000
60	FP	BoL	474.46	445.78	20.66	16.31	–0.0166	–0.0147
		EoL	478.71	497.77	19.08	12.20	–0.0184	–0.0173
		MoL	468.53	479.82	22.19	15.37	–0.0192	–0.0181
	Sal	BoL	47.37	44.04	1.80	1.67	–0.0158	–0.0138
		EoL	48.16	48.92	1.96	1.22	–0.0197	–0.0189
		MoL	46.65	47.13	1.90	1.48	–0.0202	–0.0194
90	FP	BoL	481.70	451.22	21.78	11.25	–0.0152	–0.0134
		EoL	487.98	498.22	18.97	13.51	–0.0197	–0.0189
		MoL	476.63	477.05	20.90	15.82	–0.0201	–0.0193
	Sal	BoL	47.97	44.53	1.84	1.12	–0.0145	–0.0128
		EoL	48.89	48.88	1.70	1.28	–0.0204	–0.0198
		MoL	47.55	47.01	1.76	1.53	–0.0205	–0.0198

Reference-scaled PBE criteria used if denoted by ^R^, otherwise constant-scaled criteria used.

R, reference; Sal, salmeterol; T, test.

**Table 4.** Population Bioequivalence Statistics for Impactor-Sized Mass (mcg): 100/50 mcg Strength

Flow rate (L/min)	Active	Life stage	GM (T)	GM (R)	SD (T)	SD (R)	Linearized criteria	
							Point estimate	95% Upper confidence bound
30	FP	BoL	25.73	22.91	2.10	1.71	–0.0065	0.0003
		EoL	26.76	24.27	2.24	2.50	–0.0213^R^	–0.0095
	Sal	BoL	11.00	10.24	0.83	0.76	–0.0157	–0.0112
		EoL	11.46	10.85	0.88	1.14	–0.0307^R^	–0.0200
60	FP	BoL	26.95	24.31	2.04	0.87	–0.0056	–0.0009
		EoL	27.90	25.36	2.23	1.16	–0.0074	–0.0025
	Sal	BoL	11.43	10.65	0.75	0.46	–0.0134	–0.0102
		EoL	11.81	11.07	0.94	0.54	–0.0129	–0.0090
90	FP	BoL	24.89	23.38	2.17	0.96	–0.0110	–0.0067
		EoL	26.26	24.34	1.93	1.03	–0.0115	–0.0078
	Sal	BoL	10.48	10.06	0.78	0.45	–0.0154	–0.0125
		EoL	11.03	10.50	0.69	0.54	–0.0172	–0.0147

Reference-scaled PBE criteria used if denoted by ^R^, otherwise constant-scaled criteria used.

R, reference; Sal, salmeterol; T, test.

**Table 5.** Population Bioequivalence Statistics for Impactor-Sized Mass (mcg): 250/50 mcg Strength

Flow rate (L/min)	Active	Life stage	GM (T)	GM (R)	SD (T)	SD (R)	Linearized criteria	
							Point estimate	95% Upper confidence bound
30	FP	BoL	51.30	53.12	4.72	3.80	–0.0160	–0.0118
		EoL	56.37	55.03	4.55	4.29	–0.0207	–0.0170
	Sal	BoL	8.30	9.27	0.73	0.76	–0.0073	–0.0003
		EoL	9.07	9.60	0.69	0.79	–0.0194	–0.0148
60	FP	BoL	59.52	58.00	4.50	3.02	–0.0174	–0.0147
		EoL	62.68	59.30	5.11	3.20	–0.0140	–0.0101
	Sal	BoL	9.48	9.86	0.65	0.73	–0.0203	–0.0171
		EoL	9.93	10.14	0.76	0.71	–0.0197	–0.0167
90	FP	BoL	54.57	53.43	4.24	4.53	–0.0214	–0.0179
		EoL	59.42	54.11	4.56	3.38	–0.0100	–0.0048
	Sal	BoL	8.71	9.17	0.60	0.96	–0.0268^R^	–0.0176
		EoL	9.36	9.26	0.63	0.70	–0.0218	–0.0193

Reference-scaled PBE critieria used if denoted by ^R^, otherwise constant-scaled criteria used.

R, reference; Sal, salmeterol; T, test.

**Table 6.** Population Bioequivalence Statistics for Impactor-Sized Mass (mcg): 500/50 mcg Strength

Flow rate (L/min)	Active	Life stage	GM (T)	GM (R)	SD (T)	SD (R)	Linearized criteria	
							Point estimate	95% Upper confidence bound
30	FP	BoL	100.71	93.88	7.64	25.63	–0.5291^R^	–0.3963
		EoL	110.84	106.53	10.35	13.33	–0.0452^R^	–0.0310
	Sal	BoL	8.93	8.32	0.52	2.28	–0.5391^R^	–0.4044
		EoL	9.94	9.42	0.70	1.18	–0.0480^R^	–0.0337
60	FP	BoL	118.42	117.05	9.70	6.01	–0.0170	–0.0143
		EoL	131.06	121.65	11.31	6.56	–0.0109	–0.0061
	Sal	BoL	10.19	10.10	0.61	0.52	–0.0200	–0.0184
		EoL	11.20	10.54	0.73	0.61	–0.0163	–0.0131
90	FP	BoL	123.68	117.70	9.39	6.19	–0.0156	–0.0123
		EoL	128.67	123.88	10.09	7.72	–0.0172	–0.0139
	Sal	BoL	10.39	10.06	0.59	0.57	–0.0197	–0.0176
		EoL	10.86	10.55	0.62	0.63	–0.0203	–0.0182

Reference-scaled PBE criteria used if denoted by ^R^, otherwise constant-scaled criteria used.

R, reference; Sal, salmeterol; T, test.

APSD impactor-stage data and APSD parameters are presented in Tables 7–9 and Tables 10–12, respectively. Although statistical analysis of APSD stage data and parameters (other than ISM) was not required, the APSD profiles were comparable. The highest mass in the impactor-sized fractions was, in every case, deposited in stage 3 or 4 at 30 and 60 L min^−1^ and stage 2 or 3 at 90 L min^−1^, with MMAD values for test and reference differing by ≤0.7 μm across the 36 comparisons.

**Table 7.** Aerodynamic Particle Size Distribution Mean Mass Deposition Data: 100/50 Strength

Flow rate	Life stage	Active	Product	Mass deposited, μg, mean (SD)									
				M/IP	PS	S1	S2	S3	S4	S5	S6	S7	MOC
30	BoL	FP	Test	31.8 (4.5)	34.0 (4.4)	2.7 (0.4)	6.2 (0.8)	7.8 (0.7)	7.8 (0.7)	3.2 (0.4)	0.7 (0.1)	0.1 (0.1)	0.0 (0.0)
			Ref	25.0 (2.9)	38.9 (4.3)	2.3 (0.3)	4.1 (0.4)	5.7 (0.4)	7.7 (0.6)	4.2 (0.5)	1.1 (0.2)	0.2 (0.1)	0.0 (0.0)
		Sal	Test	15.6 (2.5)	20.1 (2.5)	1.2 (0.2)	2.7 (0.3)	3.4 (0.3)	3.3 (0.3)	1.3 (0.2)	0.3 (0.1)	0.0 (0.0)	0.0 (0.0)
			Ref	12.5 (1.4)	19.8 (2.1)	1.2 (0.1)	2.1 (0.2)	2.6 (0.2)	3.2 (0.2)	1.8 (0.2)	0.5 (0.1)	0.1 (0.0)	0.0 (0.0)
	EoL	FP	Test	32.0 (4.6)	32.1 (4.3)	2.4 (0.3)	5.6 (0.8)	7.5 (0.8)	8.6 (0.7)	4.0 (0.4)	1.0 (0.1)	0.2 (0.1)	0.0 (0.0)
			Ref	25.3 (3.4)	39.3 (4.9)	2.5 (0.3)	4.6 (0.6)	6.2 (0.7)	8.0 (0.9)	4.3 (0.7)	1.1 (0.2)	0.2 (0.1)	0.0 (0.0)
		Sal	Test	15.6 (2.4)	19.4 (2.3)	1.0 (0.1)	2.4 (0.3)	3.3 (0.3)	3.7 (0.3)	1.6 (0.2)	0.4 (0.1)	0.1 (0.0)	0.0 (0.0)
			Ref	12.5 (2.2)	20.1 (2.6)	1.3 (0.2)	2.3 (0.3)	2.8 (0.3)	3.4 (0.4)	1.8 (0.3)	0.5 (0.1)	0.1 (0.0)	0.0 (0.0)
60	BoL	FP	Test	25.4 (3.5)	38.1 (4.2)	4.6 (0.6)	8.1 (0.9)	8.6 (0.7)	7.1 (0.6)	2.5 (0.3)	0.5 (0.1)	0.1 (0.1)	0.0 (0.0)
			Ref	20.8 (2.1)	42.2 (3.9)	3.7 (0.2)	5.3 (0.4)	6.8 (0.6)	7.6 (0.5)	3.4 (0.3)	0.9 (0.2)	0.2 (0.1)	0.0 (0.1)
		Sal	Test	11.8 (1.9)	22.8 (2.1)	2.1 (0.2)	3.6 (0.4)	3.7 (0.3)	2.9 (0.2)	1.0 (0.1)	0.2 (0.0)	0.0 (0.0)	0.0 (0.0)
			Ref	10.2 (1.0)	22.0 (1.2)	1.9 (0.1)	2.5 (0.2)	2.9 (0.2)	3.2 (0.2)	1.5 (0.1)	0.4 (0.2)	0.1 (0.1)	0.0 (0.0)
	EoL	FP	Test	27.7 (3.6)	37.1 (4.5)	4.4 (0.6)	7.9 (0.9)	8.8 (0.7)	7.8 (0.7)	2.8 (0.3)	0.6 (0.1)	0.1 (0.1)	0.0 (0.0)
			Ref	23.3 (2.0)	42.6 (2.5)	3.9 (0.3)	5.7 (0.3)	7.3 (0.4)	7.9 (0.5)	3.5 (0.4)	0.8 (0.1)	0.2 (0.1)	0.0 (0.0)
		SAL	Test	12.8 (1.8)	22.3 (2.2)	2.0 (0.2)	3.5 (0.3)	3.8 (0.3)	3.1 (0.3)	1.1 (0.1)	0.3 (0.0)	0.0 (0.0)	0.0 (0.0)
			Ref	11.4 (1.0)	22.1 (1.1)	2.1 (0.2)	2.7 (0.1)	3.1 (0.1)	3.3 (0.2)	1.5 (0.2)	0.4 (0.1)	0.1 (0.0)	0.0 (0.0)
90	BoL	FP	Test	26.1 (3.3)	39.2 (3.9)	6.4 (0.8)	9.3 (1.0)	8.1 (0.7)	5.6 (0.5)	1.6 (0.2)	0.3 (0.1)	0.0 (0.0)	0.0 (0.0)
			Ref	20.8 (1.6)	43.7 (2.9)	5.3 (0.3)	6.6 (0.3)	7.1 (0.3)	6.6 (0.4)	2.4 (0.3)	0.6 (0.1)	0.1 (0.1)	0.0 (0.0)
		Sal	Test	12.0 (1.7)	23.2 (2.0)	2.9 (0.3)	4.1 (0.4)	3.4 (0.3)	2.2 (0.2)	0.7 (0.1)	0.1 (0.0)	0.0 (0.0)	0.0 (0.0)
			Ref	10.2 (0.8)	22.6 (1.1)	2.7 (0.2)	3.0 (0.1)	3.0 (0.1)	2.7 (0.2)	1.0 (0.1)	0.3 (0.0)	0.0 (0.0)	0.0 (0.0)
	EoL	FP	Test	27.9 (4.2)	37.1 (3.9)	6.1 (0.6)	9.1 (0.9)	8.5 (0.7)	6.4 (0.6)	1.9 (0.2)	0.4 (0.1)	0.1 (0.1)	0.0 (0.0)
			Ref	21.6 (1.8)	44.1 (2.1)	5.5 (0.4)	6.9 (0.4)	7.4 (0.4)	6.8 (0.5)	2.5 (0.3)	0.6 (0.1)	0.1 (0.1)	0.0 (0.0)
		SAL	Test	12.6 (1.7)	22.3 (2.0)	2.8 (0.2)	4.0 (0.3)	3.5 (0.2)	2.5 (0.2)	0.8 (0.1)	0.2 (0.0)	0.0 (0.0)	0.0 (0.0)
			Ref	10.4 (0.8)	22.9 (1.0)	2.8 (0.2)	3.1 (0.2)	3.1 (0.2)	2.9 (0.2)	1.1 (0.1)	0.3 (0.1)	0.1 (0.0)	0.0 (0.0)

M/IP, mouthpiece adapter/induction port; MOC, micro-orifice collector; PS, preseparator; Ref, reference; S, stage; Sal, salmeterol.

**Table 8.** Aerodynamic Particle Size Distribution Mean Mass Deposition Data for 250/50 Strength

Flow rate	Life stage	Active	Product	Mass deposited, μg, mean (SD)									
				M/IP	PS	S1	S2	S3	S4	S5	S6	S7	MOC
30	BoL	FP	Test	61.8 (10.2)	104.2 (9.7)	4.4 (0.5)	10.3 (1.2)	14.5 (1.5)	17.2 (1.6)	7.7 (0.9)	1.6 (0.2)	0.3 (0.1)	0.0 (0.0)
			Ref	61.2 (9.4)	113.9 (9.2)	4.5 (0.6)	8.2 (0.9)	12.4 (1.2)	18.8 (1.4)	10.6 (1.2)	2.7 (0.4)	0.5 (0.1)	0.0 (0.1)
		Sal	Test	11.7 (2.1)	24.3 (2.2)	0.7 (0.1)	1.7 (0.2)	2.5 (0.3)	2.8 (0.2)	1.1 (0.1)	0.2 (0.0)	0.0 (0.0)	0.0 (0.0)
			Ref	12.0 (2.0)	23.3 (2.0)	0.9 (0.1)	1.6 (0.2)	2.3 (0.2)	3.2 (0.3)	1.7 (0.2)	0.4 (0.1)	0.1 (0.0)	0.0 (0.0)
	EoL	FP	Test	63.9 (16.3)	104.5 (8.7)	4.2 (0.5)	9.6 (1.0)	14.8 (1.1)	19.7 (2.0)	9.8 (1.3)	2.2 (0.4)	0.4 (0.1)	0.0 (0.0)
			Ref	59.3 (7.1)	115.9 (8.9)	4.9 (0.6)	8.8 (1.0)	13.0 (1.3)	19.2 (1.5)	10.8 (1.3)	2.7 (0.5)	0.5 (0.1)	0.0 (0.1)
		Sal	Test	11.8 (2.3)	24.5 (1.9)	0.7 (0.1)	1.6 (0.2)	2.6 (0.2)	3.2 (0.3)	1.4 (0.2)	0.3 (0.1)	0.0 (0.0)	0.0 (0.0)
			Ref	11.6 (1.4)	23.8 (1.9)	1.0 (0.1)	1.8 (0.2)	2.4 (0.2)	3.2 (0.3)	1.8 (0.2)	0.4 (0.1)	0.1 (0.0)	0.0 (0.0)
60	BoL	FP	Test	52.1 (6.6)	115.9 (9.7)	8.2 (1.0)	15.3 (1.4)	19.0 (1.4)	17.8 (1.8)	6.2 (0.8)	1.2 (0.3)	0.2 (0.1)	0.0 (0.0)
			Ref	53.4 (5.8)	116.1 (4.1)	7.0 (0.6)	11.1 (0.8)	16.2 (0.8)	19.4 (1.5)	8.9 (1.1)	2.1 (0.4)	0.4 (0.1)	0.0 (0.0)
		Sal	Test	9.2 (1.4)	27.3 (1.6)	1.5 (0.2)	2.7 (0.2)	3.2 (0.2)	2.6 (0.2)	0.8 (0.1)	0.2 (0.0)	0.0 (0.0)	0.0 (0.0)
			Ref	10.3 (1.2)	24.0 (0.9)	1.5 (0.1)	2.1 (0.2)	2.8 (0.2)	3.2 (0.3)	1.4 (0.2)	0.4 (0.1)	0.1 (0.0)	0.0 (0.0)
	EoL	FP	Test	50.9 (4.7)	112.4 (7.7)	7.1 (0.7)	13.7 (1.2)	19.2 (1.6)	20.3 (2.1)	7.7 (1.0)	1.6 (0.3)	0.3 (0.1)	0.0 (0.0)
			Ref	54.7 (5.4)	119.1 (4.6)	7.7 (0.7)	12.2 (1.0)	16.9 (0.9)	19.2 (1.5)	8.7 (1.2)	2.0 (0.4)	0.4 (0.1)	0.0 (0.0)
		Sal	Test	8.8 (0.9)	27.1 (1.1)	1.3 (0.1)	2.4 (0.2)	3.2 (0.3)	3.0 (0.3)	1.1 (0.1)	0.2 (0.0)	0.0 (0.0)	0.0 (0.0)
			Ref	10.5 (1.1)	24.7 (1.0)	1.6 (0.1)	2.3 (0.2)	2.9 (0.2)	3.1 (0.3)	1.4 (0.2)	0.3 (0.1)	0.1 (0.0)	0.0 (0.0)
90	BoL	FP	Test	55.5 (8.1)	111.6 (8.2)	11.7 (1.2)	18.0 (1.4)	18.3 (1.6)	13.9 (1.6)	3.9 (0.6)	0.7 (0.2)	0.1 (0.1)	0.0 (0.0)
			Ref	47.4 (3.2)	119.5 (4.7)	10.7 (1.5)	14.0 (0.9)	16.4 (1.4)	15.8 (1.9)	5.8 (0.9)	1.2 (0.3)	0.3 (0.1)	0.0 (0.0)
		Sal	Test	10.0 (1.8)	26.9 (1.7)	2.2 (0.2)	3.2 (0.2)	2.9 (0.2)	2.0 (0.2)	0.5 (0.1)	0.1 (0.0)	0.0 (0.0)	0.0 (0.0)
			Ref	9.2 (0.7)	25.3 (1.1)	2.2 (0.3)	2.6 (0.2)	2.8 (0.3)	2.6 (0.4)	1.0 (0.2)	0.2 (0.1)	0.0 (0.0)	0.0 (0.0)
	EoL	FP	Test	55.4 (8.1)	108.6 (7.8)	10.9 (1.1)	17.5 (1.3)	19.4 (1.6)	16.5 (2.0)	5.0 (0.9)	0.9 (0.3)	0.2 (0.1)	0.0 (0.0)
			Ref	48.6 (4.0)	125.2 (5.4)	11.5 (1.1)	15.0 (0.9)	16.9 (1.1)	15.4 (1.4)	5.5 (1.0)	1.1 (0.4)	0.2 (0.1)	0.0 (0.0)
		Sal	Test	9.9 (1.7)	26.5 (1.5)	2.1 (0.2)	3.1 (0.2)	3.1 (0.2)	2.3 (0.2)	0.7 (0.1)	0.1 (0.0)	0.0 (0.0)	0.0 (0.0)
			Ref	9.4 (0.8)	26.3 (1.3)	2.4 (0.2)	2.8 (0.2)	2.8 (0.2)	2.5 (0.3)	0.9 (0.2)	0.2 (0.1)	0.0 (0.0)	0.0 (0.0)

Ref, reference; S, stage; Sal, salmeterol.

**Table 9.** Aerodynamic Particle Size Distribution Mean Mass Deposition Data for 500/50 Strength

Flow rate	Lifestage	Active	Product	Mass deposited, μg, mean (SD)									
				M/IP	PS	S1	S2	S3	S4	S5	S6	S7	MOC
30	BoL	FP	Test	157.5 (24.1)	190.0 (22.7)	7.6 (0.7)	19.0 (1.2)	28.7 (2.2)	35.4 (4.3)	14.5 (1.8)	2.9 (0.5)	0.5 (0.1)	0.0 (0.0)
			Ref	109.2 (26.8)	216.4 (53.9)	7.6 (2.1)	14.5 (3.7)	24.9 (6.5)	36.9 (9.7)	18.4 (4.8)	4.2 (1.2)	0.8 (0.3)	0.1 (0.2)
		Sal	Test	15.4 (2.1)	20.9 (2.3)	0.7 (0.1)	1.8 (0.1)	2.8 (0.2)	3.2 (0.3)	1.0 (0.1)	0.2 (0.0)	0.0 (0.0)	0.0 (0.0)
			Ref	10.9 (2.7)	22.0 (5.5)	0.8 (0.2)	1.5 (0.4)	2.2 (0.6)	3.2 (0.8)	1.5 (0.4)	0.3 (0.1)	0.1 (0.0)	0.0 (0.0)
	EoL	FP	Test	149.0 (22.1)	192.9 (20.0)	7.0 (0.8)	17.8 (1.4)	29.7 (2.7)	41.1 (5.6)	18.0 (2.8)	3.9 (0.5)	0.7 (0.2)	0.0 (0.1)
			Ref	112.8 (13.6)	231.7 (29.5)	8.9 (1.1)	17.2 (2.2)	28.1 (3.6)	39.3 (5.9)	17.9 (2.8)	4.0 (0.7)	0.8 (0.2)	0.1 (0.1)
		Sal	Test	14.3 (2.2)	21.6 (1.8)	0.7 (0.1)	1.7 (0.1)	2.9 (0.2)	3.8 (0.4)	1.3 (0.1)	0.3 (0.0)	0.0 (0.0)	0.0 (0.0)
			Ref	11.1 (1.3)	23.6 (3.1)	0.9 (0.1)	1.7 (0.2)	2.5 (0.3)	3.4 (0.5)	1.5 (0.2)	0.3 (0.1)	0.0 (0.0)	0.0 (0.0)
60	BoL	FP	Test	112.0 (10.5)	221.6 (17.6)	13.3 (1.8)	28.6 (2.8)	39.3 (3.8)	35.8 (4.3)	12.0 (1.5)	2.5 (0.5)	0.5 (0.1)	0.0 (0.1)
			Ref	108.5 (6.6)	223.1 (6.6)	11.9 (1.2)	21.4 (1.7)	35.8 (2.2)	39.4 (3.3)	15.9 (1.6)	3.8 (0.6)	0.9 (0.2)	0.1 (0.1)
		Sal	Test	10.4 (1.1)	24.9 (1.1)	1.3 (0.2)	2.8 (0.2)	3.6 (0.3)	2.8 (0.2)	0.8 (0.1)	0.2 (0.0)	0.0 (0.0)	0.0 (0.0)
			Ref	10.5 (0.7)	23.0 (0.8)	1.3 (0.1)	2.0 (0.1)	3.0 (0.2)	3.4 (0.3)	1.3 (0.1)	0.3 (0.1)	0.1 (0.0)	0.0 (0.0)
	EoL	FP	Test	119.8 (13.2)	199.8 (20.0)	12.0 (1.3)	27.8 (2.1)	42.4 (3.9)	42.4 (5.0)	14.7 (1.7)	3.3 (0.5)	0.7 (0.2)	0.1 (0.1)
			Ref	106.4 (6.4)	238.8 (8.6)	13.4 (0.9)	23.9 (1.4)	37.6 (2.1)	40.1 (3.1)	15.7 (1.9)	3.7 (0.6)	0.8 (0.2)	0.1 (0.1)
		Sal	Test	11.5 (1.4)	23.1 (1.6)	1.2 (0.1)	2.7 (0.2)	4.0 (0.3)	3.3 (0.3)	1.0 (0.1)	0.2 (0.0)	0.0 (0.0)	0.0 (0.0)
			Ref	10.5 (0.6)	24.7 (1.0)	1.4 (0.1)	2.2 (0.1)	3.2 (0.2)	3.4 (0.3)	1.3 (0.2)	0.3 (0.1)	0.1 (0.0)	0.0 (0.0)
90	BoL	FP	Test	110.1 (13.2)	215.9 (19.8)	20.4 (1.9)	38.2 (2.8)	42.9 (3.7)	31.5 (3.3)	9.1 (1.0)	1.9 (0.3)	0.4 (0.1)	0.0 (0.0)
			Ref	103.4 (7.4)	220.0 (6.4)	17.8 (1.8)	29.5 (2.5)	38.2 (3.0)	34.5 (3.0)	12.0 (1.6)	2.9 (0.7)	0.7 (0.2)	0.0 (0.1)
		Sal	Test	10.3 (1.4)	24.3 (1.5)	2.1 (0.2)	3.7 (0.2)	3.7 (0.2)	2.3 (0.2)	0.6 (0.1)	0.1 (0.0)	0.0 (0.0)	0.0 (0.0)
			Ref	9.8 (0.8)	22.9 (0.8)	1.9 (0.2)	2.6 (0.2)	3.2 (0.2)	2.9 (0.3)	1.0 (0.1)	0.2 (0.1)	0.0 (0.0)	0.0 (0.0)
	EoL	FP	Test	117.2 (15.0)	204.3 (21.3)	20.0 (2.1)	38.3 (3.2)	43.7 (3.8)	33.8 (3.7)	10.5 (1.3)	2.3 (0.4)	0.5 (0.1)	0.0 (0.0)
			Ref	101.7 (7.0)	236.3 (9.1)	20.3 (1.7)	32.6 (2.6)	40.6 (2.9)	35.3 (3.0)	12.1 (1.6)	2.9 (0.6)	0.7 (0.2)	0.0 (0.1)
		Sal	Test	10.9 (1.6)	23.3 (1.7)	2.0 (0.2)	3.7 (0.3)	3.8 (0.2)	2.5 (0.2)	0.7 (0.1)	0.1 (0.0)	0.0 (0.0)	0.0 (0.0)
			Ref	9.6 (0.6)	24.6 (1.1)	2.1 (0.2)	2.9 (0.2)	3.4 (0.2)	3.0 (0.3)	1.0 (0.1)	0.2 (0.1)	0.0 (0.0)	0.0 (0.0)

Ref, reference; S, stage; Sal, salmeterol.

**Table 10.** Aerodynamic Particle Size Distribution Mean Calculated Parameters for 100/50 Strength

Flow rate	Life stage	Active	Product	MB (%), mean (SD)	ISM (μg), mean (SD)	FPM (μg), mean (SD)	MMAD (μm), mean (SD)	GSD (μm), mean (SD)
30	BoL	FP	Test	101.3 (3.3)	25.8 (2.1)	15.5 (1.5)	4.7 (0.3)	1.9 (0.0)
			Ref	95.9 (7.3)	23.0 (1.7)	15.9 (1.4)	4.0 (0.2)	2.1 (0.1)
		Sal	Test	106.5 (3.3)	11.0 (0.8)	6.5 (0.6)	4.7 (0.3)	1.9 (0.0)
			Ref	97.1 (7.5)	10.3 (0.8)	6.7 (0.6)	4.2 (0.2)	2.1 (0.1)
	EoL	FP	Test	100.3 (4.8)	26.9 (2.2)	17.5 (1.4)	4.2 (0.2)	1.9 (0.0)
			Ref	98.5 (10.5)	24.4 (2.5)	16.6 (1.9)	4.1 (0.2)	2.0 (0.1)
		Sal	Test	105.6 (3.9)	11.5 (0.9)	7.4 (0.6)	4.3 (0.2)	1.9 (0.0)
			Ref	99.6 (11.2)	10.9 (1.1)	7.0 (0.9)	4.3 (0.3)	2.1 (0.0)
60	BoL	FP	Test	102.4 (3.9)	27.0 (2.0)	20.5 (1.5)	3.9 (0.1)	2.0 (0.0)
			Ref	97.7 (4.4)	24.2 (1.2)	19.7 (1.0)	3.3 (0.1)	2.1 (0.1)
		Sal	Test	107.1 (3.4)	11.5 (0.7)	8.6 (0.6)	4.0 (0.2)	2.0 (0.0)
			Ref	99.5 (3.3)	10.6 (0.6)	8.5 (0.5)	3.5 (0.2)	2.2 (0.1)
	EoL	FP	Test	104.5 (4.1)	28.0 (2.2)	21.6 (1.7)	3.7 (0.1)	2.0 (0.0)
			Ref	102.4 (3.4)	25.4 (1.2)	20.5 (1.1)	3.4 (0.1)	2.1 (0.0)
		Sal	Test	108.9 (3.4)	11.8 (0.9)	9.0 (0.8)	3.8 (0.1)	2.0 (0.0)
			Ref	103.6 (3.4)	11.1 (0.5)	8.8 (0.5)	3.6 (0.1)	2.1 (0.0)
90	BoL	FP	Test	104.0 (3.4)	25.0 (2.2)	21.2 (1.9)	3.7 (0.1)	2.0 (0.0)
			Ref	100.2 (3.8)	23.4 (1.0)	20.8 (1.0)	3.2 (0.1)	2.1 (0.1)
		Sal	Test	108.1 (3.2)	10.5 (0.8)	8.8 (0.7)	3.8 (0.1)	2.0 (0.0)
			Ref	101.3 (3.0)	10.1 (0.4)	8.9 (0.4)	3.4 (0.1)	2.2 (0.1)
	EoL	FP	Test	104.9 (4.0)	26.3 (1.9)	22.7 (1.7)	3.5 (0.1)	2.0 (0.0)
			Ref	102.8 (3.4)	24.4 (1.0)	21.6 (1.0)	3.2 (0.1)	2.1 (0.0)
		Sal	Test	108.4 (3.0)	11.1 (0.7)	9.4 (0.6)	3.6 (0.1)	2.0 (0.0)
			Ref	103.7 (3.7)	10.5 (0.5)	9.3 (0.5)	3.4 (0.1)	2.2 (0.1)

FPM, fine particle mass (interpolated to <5 μm); GSD, geometric standard deviation; ISM, impactor-sized mass; MB, mass balance; MMAD, mass median aerodynamic diameter; Ref, reference; Sal, salmeterol.

**Table 11.** Aerodynamic Particle Size Distribution Mean Calculated Parameters for 250/50 Strength

Flow rate	Lifestage	Active	Product	MB (%), mean (SD)	ISM (μg), mean (SD)	FPM (μg), mean (SD)	MMAD (μm), mean (SD)	GSD (μm), mean (SD)
30	BoL	FP	Test	95.2 (4.4)	51.5 (4.7)	33.9 (3.2)	4.2 (0.1)	1.9 (0.0)
			Ref	99.9 (7.0)	53.2 (3.8)	38.7 (3.1)	3.7 (0.2)	2.0 (0.0)
		Sal	Test	100.1 (3.8)	8.3 (0.7)	5.3 (0.5)	4.4 (0.1)	1.8 (0.0)
			Ref	101.2 (7.5)	9.3 (0.8)	6.5 (0.7)	3.9 (0.2)	2.0 (0.1)
	EoL	FP	Test	98.4 (6.7)	56.6 (4.6)	39.6 (3.8)	3.9 (0.2)	1.9 (0.0)
			Ref	101.0 (6.7)	55.2 (4.3)	39.7 (3.4)	3.7 (0.2)	2.0 (0.0)
		Sal	Test	102.3 (3.8)	9.1 (0.7)	6.2 (0.6)	4.1 (0.2)	1.9 (0.0)
			Ref	102.3 (7.2)	9.6 (0.8)	6.6 (0.7)	4.0 (0.2)	2.1 (0.1)
60	BoL	FP	Test	101.2 (4.7)	59.7 (4.5)	47.1 (4.0)	3.6 (0.1)	1.9 (0.0)
			Ref	100.7 (2.7)	58.1 (3.0)	49.2 (4.0)	3.1 (0.2)	2.0 (0.1)
		Sal	Test	105.5 (3.7)	9.5 (0.7)	7.3 (0.5)	3.7 (0.1)	2.0 (0.0)
			Ref	101.5 (2.8)	9.9 (0.7)	8.1 (0.7)	3.3 (0.2)	2.1 (0.0)
	EoL	FP	Test	100.1 (3.7)	62.9 (5.1)	52.6 (5.3)	3.3 (0.2)	1.9 (0.1)
			Ref	103.3 (3.3)	59.4 (3.2)	49.3 (3.7)	3.2 (0.2)	2.0 (0.1)
		Sal	Test	104.7 (3.4)	10.0 (0.8)	7.9 (0.7)	3.5 (0.1)	1.9 (0.0)
			Ref	104.3 (3.7)	10.2 (0.7)	8.2 (0.7)	3.4 (0.2)	2.1 (0.0)
90	BoL	FP	Test	100.2 (3.8)	54.7 (4.2)	47.5 (4.0)	3.4 (0.2)	2.0 (0.0)
			Ref	99.2 (3.0)	53.6 (4.5)	48.1 (4.5)	3.1 (0.1)	2.1 (0.1)
		Sal	Test	106.3 (3.2)	8.7 (0.6)	7.4 (0.5)	3.7 (0.1)	2.0 (0.0)
			Ref	102.1 (3.0)	9.2 (1.0)	8.2 (0.9)	3.3 (0.2)	2.2 (0.1)
	EoL	FP	Test	100.6 (3.1)	59.6 (4.6)	52.7 (4.5)	3.2 (0.2)	2.0 (0.0)
			Ref	102.8 (3.6)	54.2 (3.4)	48.2 (3.4)	3.2 (0.1)	2.1 (0.1)
		Sal	Test	106.3 (2.6)	9.4 (0.6)	8.1 (0.6)	3.4 (0.2)	2.0 (0.0)
			Ref	105.3 (3.7)	9.3 (0.7)	8.1 (0.7)	3.4 (0.2)	2.2 (0.1)

Ref, reference; Sal, salmeterol; Test, Wixela Inhub.

**Table 12.** Aerodynamic Particle Size Distribution Mean Calculated Parameters for 500/50 Strength

Flow rate	Lifestage	Active	Product	MB (%), mean (SD)	ISM (μg), mean (SD)	FPM (μg), mean (SD)	MMAD (μm), mean (SD)	GSD (μm), mean (SD)
30	BoL	FP	Test	98.1 (3.5)	101.0 (7.6)	67.4 (6.7)	4.1 (0.2)	1.8 (0.0)
			Ref	93.1 (23.0)	99.8 (25.6)	72.8 (18.9)	3.7 (0.1)	1.9 (0.0)
		Sal	Test	102.0 (2.1)	8.9 (0.5)	5.7 (0.4)	4.4 (0.1)	1.8 (0.0)
			Ref	94.5 (23.4)	8.8 (2.3)	6.2 (1.6)	3.9 (0.2)	1.9 (0.0)
	EoL	FP	Test	99.0 (3.9)	111.3 (10.3)	78.6 (9.1)	3.9 (0.1)	1.8 (0.0)
			Ref	99.1 (11.7)	107.4 (13.3)	75.9 (10.1)	3.9 (0.1)	1.9 (0.0)
		Sal	Test	103.4 (3.1)	10.0 (0.7)	6.8 (0.6)	4.1 (0.1)	1.8 (0.0)
			Ref	100.4 (12.1)	9.5 (1.2)	6.5 (0.9)	4.1 (0.1)	1.9 (0.0)
60	BoL	FP	Test	100.1 (3.6)	118.8 (9.7)	96.9 (10.4)	3.4 (0.2)	1.9 (0.1)
			Ref	99.1 (2.0)	117.2 (6.0)	102.3 (7.2)	3.0 (0.2)	1.9 (0.1)
		Sal	Test	104.1 (3.1)	10.2 (0.6)	8.0 (0.5)	3.7 (0.1)	1.9 (0.1)
			Ref	99.8 (2.4)	10.1 (0.5)	8.4 (0.6)	3.2 (0.2)	2.0 (0.1)
	EoL	FP	Test	99.6 (3.5)	131.5 (11.3)	112.6 (11.1)	3.2 (0.1)	1.8 (0.0)
			Ref	103.3 (3.3)	121.8 (6.6)	104.5 (8.0)	3.1 (0.1)	1.9 (0.1)
		Sal	Test	104.4 (3.2)	11.2 (0.7)	9.2 (0.8)	3.4 (0.1)	1.8 (0.1)
			Ref	104.7 (3.2)	10.6 (0.6)	8.6 (0.6)	3.3 (0.1)	2.0 (0.0)
90	BoL	FP	Test	101.2 (3.3)	124.0 (9.4)	109.3 (8.8)	3.2 (0.1)	1.9 (0.0)
			Ref	98.7 (2.0)	117.9 (6.2)	106.7 (6.2)	2.9 (0.1)	2.0 (0.0)
		Sal	Test	104.7 (3.0)	10.4 (0.6)	8.9 (0.5)	3.5 (0.1)	1.9 (0.0)
			Ref	99.3 (2.2)	10.1 (0.6)	9.0 (0.6)	3.1 (0.1)	2.0 (0.0)
	EoL	FP	Test	101.2 (3.2)	129.1 (10.1)	114.4 (9.5)	3.1 (0.1)	1.9 (0.0)
			Ref	103.7 (3.9)	124.1 (7.7)	111.6 (7.4)	3.0 (0.1)	2.0 (0.0)
		Sal	Test	104.7 (2.4)	10.9 (0.6)	9.4 (0.6)	3.4 (0.1)	1.9 (0.0)
			Ref	104.2 (3.7)	10.6 (0.6)	9.4 (0.6)	3.2 (0.1)	2.0 (0.1)

Ref, reference; Sal, salmeterol.

The PBE comparisons for SAC and ISM, together with the broad comparability of APSD, demonstrated acceptable *in vitro* equivalence and were supportive of the overall finding of BE between the two products.

#### Airflow resistance

The airflow resistance of Inhub and Diskus is presented in Table 13. The value of 0.027 kPa^0.5^/L min^−1^ obtained for Diskus is highly comparable with the majority of previous reports of its resistance.^(12)^ The resistance of Inhub at 0.034 kPa^0.5^ L min^−1^ was found to be ∼30% higher compared with Diskus. Table 13 also shows that the resistance of Inhub was found to be more consistent compared with Diskus, showing less than half the variability of Diskus when measured across multiple batches. Thus, the flow rate at 4 kPa pressure drop through Diskus ranged from 67 to 89 L min^−1^, whereas that through Inhub showed a much narrower range, of 56 to 60 L min^−1^.

**Table 13.** Airflow Resistance of the Diskus and Inhub Devices

	Diskus	Inhub
Specific airflow resistance, kPa^0.5^ L min^−1^	0.027	0.034
Pressure drop at 60 L min^−1^, kPa		
Mean	2.6	4.5
Range	2.1–3.4	4.1–4.8
RSD	9%	4%
Flow rate at 4 kPa pressure drop, L min^−1^		
Mean	75.3	58.4
Range	67.2–89.1	56.3–59.8
RSD	7%	2%

RSD, relative standard deviation.

#### ED and ISM at fixed pressure drop

The ED and ISM at fixed pressure drop of 4 kPa were characterized for each strength of the two products, and are also shown in Figures 3 and 4 (data shown as open circles). For Inhub, the flow rate at 4 kPa was consistently close to 60 L min^−1^ (Table 13) and the *in vitro* performance at 4 kPa was similar to that measured at 60 L min^−1^. For Diskus, the mean flow rate at 4 kPa was 75 L min^−1^. As expected, the *in vitro* performance at 4 kPa was similar to that measured at 60 or 90 L min^−1^, with little difference in performance across this flow rate range.

### Patient inhalation flow profile study

#### Patients

The study was conducted from August through December 2013 at four study sites in Germany. Of 93 subjects (across the five study groups) screened, 80 were eligible for the study and performed the flow profiling study; 78 subjects were included in the inhalation analysis set as one subject from each of the 4–7 and 8–11-year groups were excluded due to inclusion/exclusion criteria deviations discovered after randomization. Subjects were randomized to manage the order of the flow profiling through each inhaler to ensure no bias was associated with the order of testing the inhalers. Figure 2 shows a diagram of subject disposition. Baseline characteristics of subjects are shown in Table 14. Overall, 30 (37.5%) randomized subjects were female.

**Table 14.** Subject Demographics and Clinical Characteristics in the Inhalation Flow Profile Study

Characteristic	Healthy adults (*n* = 10)	Children with asthma ages 4–7 years (*n* = 21)	Children with asthma ages 8–11 years (*n* = 21)	Adults with asthma (*n* = 14)	Adults with severe COPD (*n* = 14)
Age, years					
Mean (SD)	33.1 (9.4)	5.6 (1.1)	9.7 (1.2)	55.9 (7.1)	64.4 (7.29)
Range	20–49	4–7	8–11	40–64	51–78
Sex, *n* (%)					
Male	6 (60)	14 (66.7)	16 (76.2)	5 (35.7)	9 (64.3)
Female	4 (40)	7 (33.3)	5 (23.8)	9 (64.3)	5 (35.7)
Height, cm	173.7 (8.7)	118.8 (7.7)	144.3 (12.0)	167.6 (9.7)	168.4 (8.7)
Mean (SD)					
Weight, kg, mean (SD)	72.3 (14.4)	22.2 (3.7)	39.1 (14.1)	89.6 (23.7)	74.2 (11.7)
Body mass index, kg/m^2^, mean (SD)	23.8 (3.3)	15.6 (1.3)	18.2 (3.2)	31.9 (8.3)	26.3 (5.3)
FEV_1_, % of predicted					
Mean (SD)	107.7 (11.2)	93.2 (14.1)	86.7 (9.2)	54.3 (9.8)	40.0 (5.8)
Range	88.7–127.1	64.6–117.0	68.6–108.0	30.1–71.0	28.0–48.1
Smoking history, *n* (%)					
Nonsmoker	8 (80)	21 (100)	21 (100)	9 (64.3)	0
Ex-smoker	2 (20)	0	0	4 (28.6)	9 (64.3)
Current smoker	0	0	0	1 (7.1)	5 (35.7)
Smoking history, pack-years	4.8 (5.3)	N/A	N/A	4.6 (3.4)	42.6 (21.5)
Mean (SD)					

COPD, chronic obstructive pulmonary disease; FEV_1_, forced expiratory volume in 1 second; N/A, not applicable.

#### Inhalation profile results

**FIG. 5.**
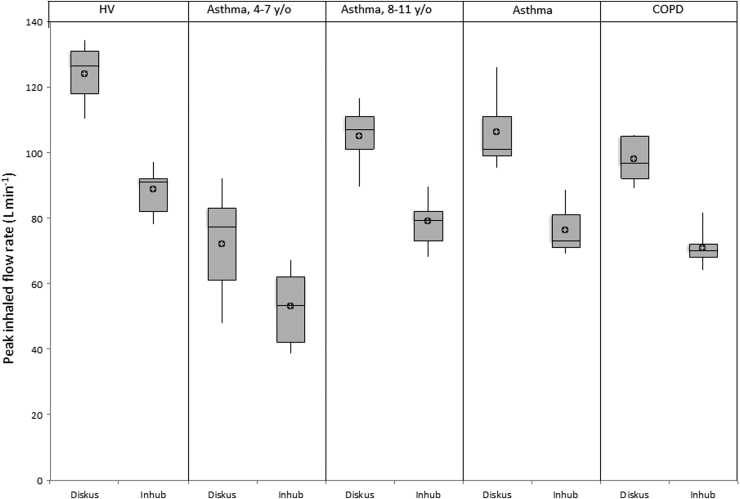
Mean peak inhaled flow rate by subject group for Wixela Inhub and Advair Diskus. To generate each inhalation profile, subjects inhaled through the dry-powder inhaler within the connecting box through a disposable filter. Before the inhalation profiles were obtained, subjects were trained to inhale rapidly and deeply using both the Inhub and Diskus devices. After the training inhalations, each subject was asked to generate three inhalation profiles through one device, followed by three inhalation profiles through the other device. HV, healthy volunteers; asthma 4–7 y/o, children (4–7 years old) with asthma; asthma 8–11 y/o, children (8–11 years old) with asthma; asthma, participants (18–80 years old) with asthma; COPD, participants (40–80 years old) with severe chronic obstructive pulmonary disease; Diskus, Advair Diskus; Inhub, Wixela Inhub. Open circles represent mean values; boxes represent the first quartile, median, and third quartile; vertical lines represent the minimum and maximum values in the range.

The results of the PIFR analyses are presented in Table 15 and Figure 5. Among the five study groups, PIFR was lowest in children with asthma 4 to 7 years of age and highest in healthy adults for both devices. Among adult subjects, those with severe COPD produced the lowest PIFR values. Overall, the mean and “best of 3” PIFR values were comparable within each of the subject groups for both Inhub and Diskus. As was expected based on the slightly higher resistance of the Inhub, higher PIFRs were measured for Diskus compared with Inhub.

**Table 15.** Summary of Peak Inhaled Flow Rate Results

	Healthy adults (*n* = 10)	Children with asthma ages 4–7 years (*n* = 20)	Children with asthma ages 8–11 years (*n* = 20)	Adults with asthma (*n* = 14)	Adults with severe COPD (*n* = 14)
PIFR (L min^−1^)					
Mean values					
Inhub					
*n*	10	20	20	14	14
Mean (SD)	86.3 (7.58)	50.6 (11.13)	75.4 (9.53)	74.8 (8.99)	69.5 (6.36)
Diskus					
*n*	10	20	20	14	14
Mean (SD)	120.7 (9.48)	66.8 (16.32)	100.6 (10.07)	102.9 (12.75)	94.9 (8.96)
PIFR (L min^−1^)					
Best of 3					
Inhub					
*n*	10	20	20	14	14
Mean (SD)	88.6 (7.24)	53.2 (10.85)	79.1 (7.79)	76.5 (9.06)	70.8 (6.65)
Diskus					
*n*	10	20	20	14	14
Mean (SD)	124.1 (9.11)	72.0 (16.48)	105.2 (9.59)	106.5 (13.03)	98.2 (8.63)

PIFR, peak inhaled flow rate.

The results of the peak pressure drop analyses are presented in Table 16. Among the five study groups, peak pressure drop was lowest in children with asthma 4 to 7 years of age and highest in healthy adults for both devices. Among adult subjects, those with severe COPD produced the lowest peak pressure drop values. Overall, the mean and “best of 3” peak pressure drop values were comparable within each of the subject groups for both Inhub and Diskus.

**Table 16.** Summary of Peak Pressure Drop Analyses

	Healthy adults (*n* = 10)	Children with asthma ages 4–7 years (*n* = 20)	Children with asthma ages 8–11 years (*n* = 20)	Adults with asthma (*n* = 14)	Adults with severe COPD (*n* = 14)
Pressure drop (kPa)					
Mean					
Inhub					
*n*	10	20	20	14	14
Mean (SD)	9.14 (1.58)	3.27 (1.40)	7.05 (1.62)	6.90 (1.75)	5.93 (1.13)
Diskus					
*n*	10	20	20	14	14
Mean (SD)	8.58 (1.33)	2.78 (1.23)	5.99 (1.13)	6.30 (1.67)	5.39 (0.99)
Pressure drop (kPa)					
Best of 3					
Inhub					
*n*	10	20	20	14	14
Mean (SD)	9.62 (1.55)	3.58 (1.42)	7.69 (1.50)	7.22 (1.79)	6.15 (1.20)
Diskus					
*n*	10	20	20	14	14
Mean (SD)	9.06 (1.31)	3.18 (1.33)	6.53 (1.15)	6.73 (1.75)	5.73 (1.03)

## Discussion

The *in vitro* studies of ED and ISM demonstrated the equivalence of the Wixela Inhub and Advair Diskus products at 30, 60, and 90 L min^−1^. These data formed a key part of the overall dataset required to establish BE between the innovator and subsequent-entry products as required by FDA guidance.

The patient inhalation flow profile study was conducted to measure the flow rates achieved across the range of patient populations for which Wixela Inhub is indicated. The study compared PIFR and peak pressure drop for healthy adults, children with asthma, and adults with asthma or severe COPD when they inhaled through the Inhub or Diskus devices.

The flow rates achieved in the *in vivo* flow profiling study were in line with expectations, considering the resistance of the two devices. In healthy subjects and in all patient groups, slightly lower PIFRs were achieved through the Inhub device than through Diskus. Despite the slightly higher resistance of Inhub, all patients achieved PIFRs >30 L min^−1^; that is, all PIFRs exceeded the flow rate tested in the *in vitro* studies. The minimum PIFR obtained from children 4 to 7 years of age was 38.0 L min^−1^, and all other study subjects generated PIFRs ≥65.0 L min^−1^, indicating that adult patients and children with asthma or COPD will be able to generate sufficient inspiratory force to receive an acceptable dose based on the *in vitro* equivalence of Wixela Inhub and Advair Diskus demonstrated at 30, 60, and 90 L min^−1^ flow rates.

The PIFRs generated with Diskus in the current study are similar to those previously reported for children with asthma and adults with asthma or severe COPD, which confirms the validity of the current study.^(2,15–17,19,20)^ In a previous study, children with asthma generated PIFRs through Diskus ranging from 57 to 121 L min^−1^.^(21)^ In our study, the range of PIFRs generated by children through Diskus was 30 to 116 L min^−1^. In another previously published study, adults with COPD or asthma generated PIFRs through Diskus >90 L min^−1^.^(19)^ Additional studies of Diskus have reported mean PIFRs of 82 L min^−1^ for adults with COPD and 122 L min^−1^ for adolescents with asthma.^(2)^ In the current study, the mean PIFRs generated through the Diskus by adults with asthma and severe COPD were >90 L min^−1^.

In our inhalation flow profile study, healthy adults generated PIFR values greater than those generated by adult patients with asthma and COPD for both Inhub and Diskus; however, the Inhub:Diskus ratio was comparable across the patient groups. As both inhalers are relatively insensitive to changes in inhaled flow rates, it can therefore be anticipated that the ability to inhale through the Inhub would be similar regardless of disease status, and thus, healthy subjects were appropriate to utilize in the PK BE studies. These findings further support the current requirement to use healthy subjects in PK studies per the FDA guidance.

The differences in PIFRs between Inhub and Diskus are not considered clinically significant, because of the low flow-rate dependency of product performance observed in the clinically relevant flow-rate ranges for each product strength in the *in vitro* studies. Wixela Inhub showed minimal *in vitro* flow dependency of SAC and of ISM in the 100/50 mcg and 250/50 mcg strengths. Advair Diskus showed generally similar flow dependency in the bulk of the data, although the mean values at 30 L min^−1^ (particularly for SAC) were negatively impacted by a significant subpopulation of doses giving low-dose emission at this flow rate. This observation has no clinical significance, because all patient groups achieved flow rates well over 30 L min^−1^ from Diskus. Both products showed more appreciable flow dependency of ISM between 30 and 60 L min^−1^ in the 500/50 mcg strength. However, minimal flow dependency was observed between 60 and 90 L min^−1^, and all adult patients with asthma (for whom the 500/50 mcg strength is indicated) achieved >60 L min^−1^ flow rate for both products.

Whereas Inhub has a slightly higher airflow resistance (0.034 kPa^0.5^ L min^−1^) than Diskus (0.027 kPa^0.5^ L min^−1^), it can be regarded as comparable. Similar *in vitro* performance was obtained from the two products at a fixed pressure drop. The difference in resistance was not evident to participants in clinical trials. The differences in PIFRs had little or no impact on the successful demonstration of clinical and PK BE between Advair Diskus and Wixela Inhub at each dose strength.^(7,8)^

Van der Palen et al. reported that patients generally report increased acceptability with decreased device resistance, but that reaches a plateau at a resistance of ∼0.063 kPa^0.5^ minutes L^−1^.^(22)^ Because both Inhub and Diskus have a resistance below this threshold, it is unlikely to play a role in patient preference between the two devices. In addition, Inhub has been shown in our *in vitro* studies to have more reproducible resistance compared with Diskus, which may contribute to a more consistent user experience with Inhub.

Based on the airflow resistance of 0.034 kPa^0.5^ L min^−1^ determined for Inhub in these studies, the “medium” resistance preset would be selected if using the In-Check DIAL G16 to check inhaler technique in relation to Inhub use, because the Inhub resistance falls within the reported range for the devices currently assigned to this category by the manufacturers of In-Check.^(12)^

A recent publication from Clark et al. suggested that the focus of assessing whether a patient would receive an adequate lung dose from any given DPI should be based on the negative pressure generated by the patient's inspiratory effort rather than focusing on PIFR.^(23)^ A pressure drop of >1 kPa was suggested as a reasonable alternative threshold above which a patient should receive an adequate lung dose from a DPI.^(21)^ In the patient inhalation flow profile study, the pressure drops for Inhub were similar to those of Diskus, and importantly, all patients achieved a pressure drop of >1 kPa with the Inhub. This suggests that regardless of whether PIFR or pressure drop is considered the most suitable assessment of whether a patient would likely receive an adequate lung dose, patients would be able to achieve this with Inhub.

The *in vitro* studies reported here confirm that inspiratory flow >30 L min^−1^ is sufficient for Inhub use, and the results of the flow profile studies suggest that all patient groups will be able to achieve this if following the instructions for use.

## Conclusion

Results of the *in vitro* studies indicate that the Inhub device has slightly greater airflow resistance than the Diskus, but that Wixela Inhub performs consistently and comparably to Advair Diskus with respect to *in vitro* dose delivery characteristics, across all product strengths and at a range of flow rates that can be achieved by the target population. Results from the patient inhalation flow study demonstrate that healthy subjects and patients with asthma or COPD are able to generate PIFRs through the Inhub that exceeded the minimum flow rate tested in the *in vitro* studies (≥30 L min^−1^). The results from the *in vitro* BE studies reported here, together with the PK and clinical BE reported elsewhere,^(7,8)^ demonstrate that the difference in resistance did not impact the overall demonstration of BE of the products.

The combination of the *in vitro* equivalence as described in this study and the *in vivo* PK and local therapeutic equivalence (reported separately)^(7,8)^ confirm that Wixela Inhub is a substitutable generic equivalent to Advair Diskus across all indicated patient groups, including pediatric patients with asthma and adults with asthma or COPD.

## Authorship

All authors, including the coauthors, are responsible for a significant part of the article. All authors and coauthors have contributed to writing the article, reviewing it, and revising its intellectual and technical content. Any author whose name appears on this article assumes responsibility and accountability for the results.

## Notes

Reviewed by:

Hak-Kim Chan

Michael Hindle
